# Heightened BTK-dependent cell proliferation in unmutated chronic lymphocytic leukemia confers increased sensitivity to ibrutinib

**DOI:** 10.18632/oncotarget.6727

**Published:** 2015-12-22

**Authors:** Ailin Guo, Pin Lu, Natalie Galanina, Chadi Nabhan, Sonali M. Smith, Morton Coleman, Y. Lynn Wang

**Affiliations:** ^1^ Division of Genomic and Molecular Pathology, Department of Pathology, University of Chicago, Chicago, IL, USA; ^2^ Department of Medicine, Section of Hematology and Oncology, University of Chicago, Chicago, IL, USA; ^3^ Department of Medicine, Weill Cornell Medical College, New York, NY, USA

**Keywords:** BTK, ibrutinib, CLL proliferation, BCR pathway, IGHV mutational status

## Abstract

In chronic lymphocytic leukemia (CLL), patients with unmutated immunoglobulin heavy chain variable region gene (UM-CLL) have worse outcomes than mutated CLL (M-CLL) following chemotherapy or chemoimmunotherapy. However, in the era of BCR-targeted therapies, the adverse prognostic impact of unmutated IGHV seems to be diminishing, and there are clinical datasets showing unexpected improved responses in UM-CLL. We investigated the biological differences of BTK activity between these subgroups and further compared the impact of ibrutinib on molecular and cellular behaviors. Immunoblotting analysis revealed that phosphorylated active BTK is significantly higher in UM-CLL. Moreover, UM-CLL, compared to M-CLL, displayed a much higher proliferative capacity that was correlated with higher phospho-BTK and greater sensitivity to ibrutinib. In addition, BTK depletion with siRNA led to a more prominent reduction in the proliferation of UM-CLL, suggesting that elevated BTK activity is responsible for increased cell proliferation. Further, cell signaling activity by multiple measurements was consistently higher in UM-CLL accompanied by a higher sensitivity to ibrutinib. These studies link UM-CLL to elevated BCR signaling, heightened BTK-dependent cell proliferation and increased sensitivity to ibrutinib. The prognostic significance of IGHV mutation should be reevaluated in the era of new therapies targeting BCR signaling.

## INTRODUCTION

Chronic lymphocytic leukemia (CLL) is the most common leukemia in Western countries and is characterized by the accumulation of CD5+ monoclonal B cells in peripheral blood and secondary lymphoid tissues. Although the morphology and immunophenotypical features are shared among CLL tumor cells, the clinical course of the disease varies from indolent to aggressive related to a variety of established prognostic factors such as age, Rai stage, chromosomal abnormalities, mutational status of the immunoglobulin heavy chain gene variable region (IGHV), ZAP70 and CD38 expression [1]. Among these, IGHV mutational status is of particular importance. Unmutated IGHV, defined as > 98% match in DNA sequences to the germline sequences, is associated with an inferior clinical course compared to mutated IGHV (< 98% homology) [2, 3]. On multivariate analyses, IGHV mutation stands as an independent prognostic factor that predicts time-to-treatment and overall survival [1, 4]. Patients with unmutated IGHV, in general, have a more aggressive disease course requiring earlier therapeutic intervention while those with mutated IGHV have much slower disease progression [2, 3]. The median overall survival for early stage patients with unmutated IGHV is 12.6 years as opposed to 23.3 years in those with mutated IGHV [4]. For patients receiving chemotherapy or chemoimmunotherapy, unmutated IGHV remains an inferior prognostic indicator. [5–7]. A long-term analysis showed significant differences between UM-CLL and M-CLL in terms of progression-free survival (33 months *vs*. 52 months, *p* = 0.01) and overall survival (78 months *vs*. not reached, *p* = 0.01) following fludarabine and rituximab chemoimmunotherapy [5]. Thus, IGHV mutation status is a clinically relevant prognostic marker in CLL.

Functionally, the IGH chain is a key component of the multimeric B-cell receptor (BCR) complex that is responsible for antigenic recognition at the surface of normal B cells. Antigen binding and BCR cross-linking triggers the activation of proximal tyrosine kinases LYN, SYK, and subsequently BTK and PI3K. The BCR signaling cascade leads to intracellular calcium release, activation of AKT and MAP kinase pathways, and nuclear translocation of NF-κB. These signaling activities culminate in increased B cell survival, proliferation and differentiation [8].

BCR signaling activity is aberrantly higher in CLL than that of normal mature B cells [9], and deregulated BCR-signaling is considered a critical driving pathologic mechanism leading to CLL development, disease progression and relapse. Several BCR-targeted agents, including inhibitors of BTK (ibrutinib), PI3Kδ (idelalisib) and SYK (R406/fostamatinib) have demonstrated not only promising preclinical activities [9–18] but also remarkable clinical efficacy against CLL in large clinical trials [19–23]. These data led to recent accelerated FDA approval of both ibrutinib and idelalisib for the treatment of relapsed and refractory CLL, and ibrutinib in 17p-deleted high-risk CLL in both treatment-naïve and relapsed settings.

Interestingly, between the two CLL subgroups with distinct IGHV mutational status, *in vitro* responses to surface Ig ligation and subsequent BCR signaling capacity are different. The majority of UM-CLL cases respond to B-cell receptor ligation while most M-CLL show no response as demonstrated by several groups with multiple different assays including global protein tyrosine phosphorylation, gene expression profiling, cellular metabolic activity, apoptotic response and proliferative activity [24–27].

Based on these *in vitro* findings, it is reasonable to speculate that CLL patients with UM IGHV would respond well to BCR-targeted therapy. Data presented in several recent clinical studies suggest that, in patients treated with ibrutinib or idelalisib, the gaps in progression free and overall survival between UM and M subgroups have diminished [20, 28]. In contrast to chemoimmunotherapy trials, the outcomes of UM-CLL and M-CLL show nearly overlapping outcomes.

In addition to narrowed differences in survival, there are even suggestions that UM-CLL may be more responsive than M-CLL to the newer therapies by certain measures. The pivotal trial leading to ibrutinib's approval for clinical use in the relapsed and refractory CLL population showed an overall ibrutinib response rate of 70% (with 20% additional patients achieving a partial response with peripheral lymphocytosis). Notably, in subset analyses, responses did not differ based on age, initial Rai stage, previous number of chemotherapy regimens, presence of del (17p)/del (11q) and levels of serum b2-microglobulin. However, patients with unmutated IGHV displayed a significantly higher overall response rate (77%) than patients with mutated IGHV (33%, *p* = 0.005) [20]. This clinical observation was preserved in a subsequent study of ibrutinib in the elderly patients where the overall response rate in unmutated group was 86.7% *vs* 56.3% in mutated [23]. Additionally, in the study comparing idelalisib + rituximab vs rituximab, it was shown that the unmutated group has a hazard ratio (HR) of 0.13 for disease progression/death versus an HR of 0.25 in the mutated group, suggesting the UM-CLL group has a lower risk of disease progression [22]. Moreover, after 3 years of treatment, the quality of response appears remarkably higher in treatment-naïve patients with UM-CLL (40% complete remission) compared to 6% in M-CLL. ([29], Supplementary Table 3). These findings have suggested that the UM group may no longer do worse than M-CLL, although not by all clinical measurements. As ibrutinib and other BCR-directed therapies are being rapidly incorporated into CLL treatment armamentarium, understanding how UM and M-CLL differ biologically and whether ibrutinib perturbs these cells in similar or different manners may be clinically relevant.

## RESULTS

### Unmutated CLL has higher BTK activity (p-BTK) than mutated CLL

**Figure 1:**
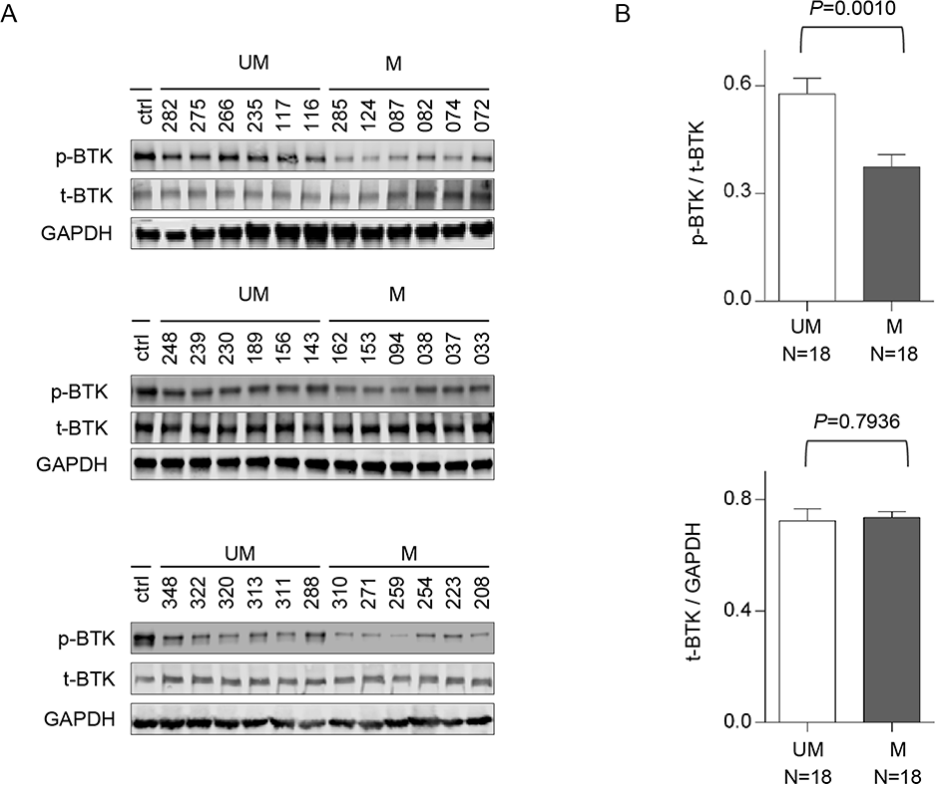
Unmutated CLL has higher levels of phosphorylated BTK than mutated CLL CD5+/CD19+ CLL-B cells were isolated from unmutated CLL patients (*N* = 18) and mutated CLL patients (*N* = 18). (**A**) The whole cell lysates were analyzed by immunoblotting with antibodies against either total BTK or phosphorylated BTK (pY223). DLBCL cell line HBL1 was used as a control for the expression of t-BTK and p-BTK. (**B**) The amounts of protein were quantified with grayscale scan. Total BTK was used to normalize p-BTK and GAPDH was used to normalize total BTK. Paired *t* test was used to analyze the data and results are shown as mean ± SEM. UM, unmutated CLL; M, mutated CLL.

Based on our previous observation that the active phosphorylated form of BTK (Y223), but not total BTK, was significantly higher in CLL B-cells than in normal B cells [9], we conjectured that greater BTK activity may underlie the better clinical responses of UM-CLL subgroup to ibrutinib. To test this hypothesis, we compared the levels of p-BTK (Y223) in UM-CLL (*n* = 18) versus M-CLL (*n* = 18). By immunoblot analyses, Figure 1A showed that the p-BTK (Y223) levels were higher in UM-CLL samples than those in M-CLL, while the amount of total-BTK was comparable between the two groups. The levels of p-BTK (Y223) and total BTK were quantified and normalized (Figure 1B), the difference in p-BTK (Y223) between the two groups was statistically significant while no difference was observed with respect to total BTK.

### UM-CLL is more proliferative than M-CLL in two different *in vitro* CLL proliferation models

**Figure 2:**
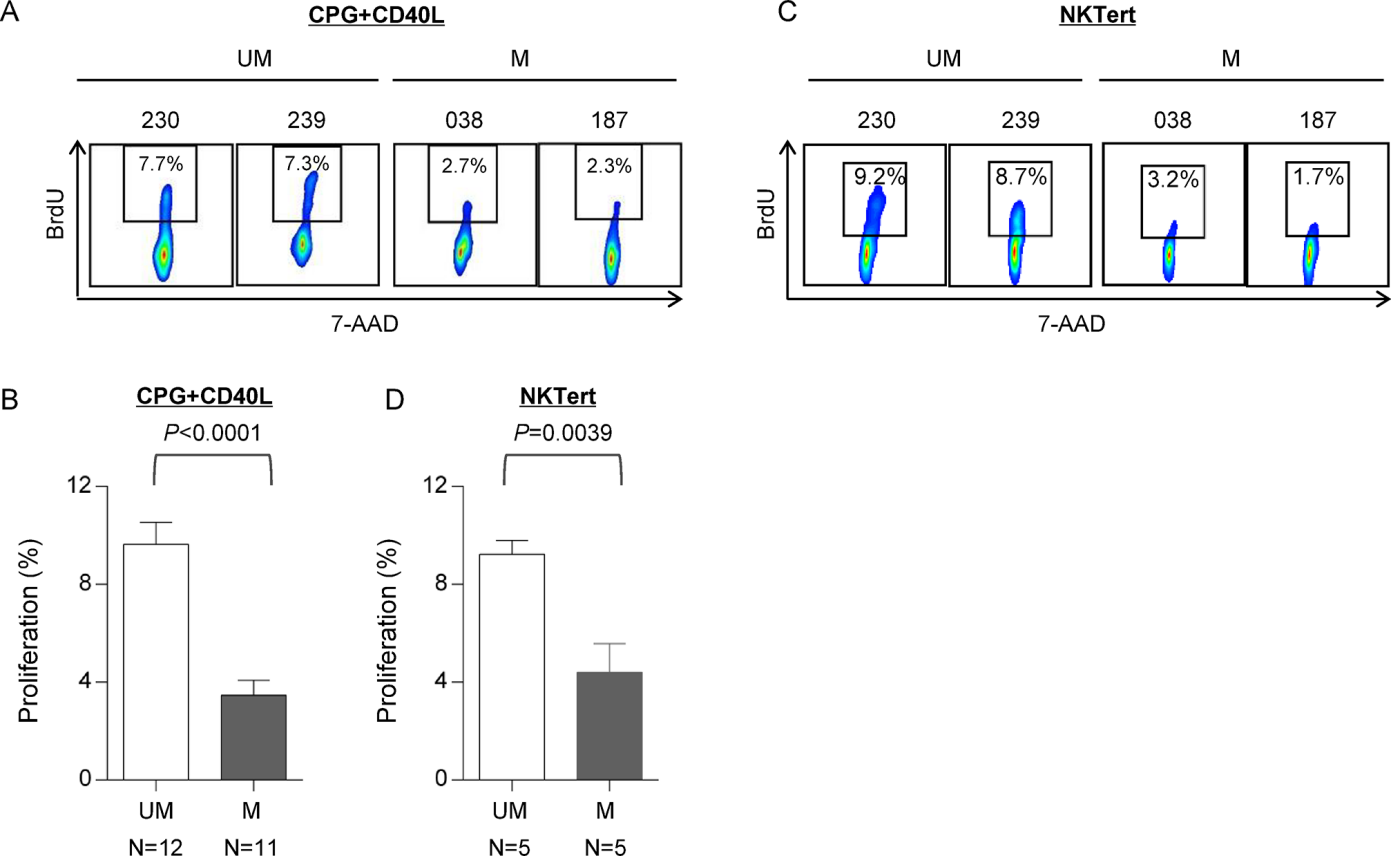
UM-CLL is more proliferative than M-CLL in both CpG + CD40L and NKTert stromal coculture models (**A** and **B**) CpG + CD40L-induced proliferation in CLL. BrdU incorporation was measured following 8 days of culture. (A) Two representative UM-CLL and M-CLL cases are shown. (B) Analysis of the aggregate data of 12 UM-CLL and 11 M-CLL cases. (**C** and **D**) Stroma coculture-induced proliferation in CLL. BrdU incorporation was measured following 20 days of culture. (C) Two representative UM-CLL and M-CLL cases are shown. (D) Analysis of the aggregate data of 5 UM-CLL and 5 M-CLL cases. Paired *t* test was used to analyze the data. Values in bar graphs represent means ± SEM. UM, unmutated CLL. M, mutated CLL.

Our group and others have demonstrated that CLL proliferation plays an important role in the pathogenesis of CLL [10, 25, 30, 31]; changes in CLL proliferation directly correlate with patient's disease activity; and anti-proliferation is one of the major therapeutic effects of ibrutinib [9, 10, 32, 33]. CLL proliferation diminishes during remission and increases with disease progression [9, 32, 33]. We thus postulated that UM-CLL has higher proliferative capacity than M-CLL. To demonstrate this, we assessed CLL proliferation in two *in vitro* proliferation models. In the first model, CLL cells were stimulated with TLR9 agonist CpG and CD40L [34–36] followed by measurements of BrdU incorporation after an 8-day culture to assess cell proliferative capacity. In Figure 2A, two representative cases are shown from each of the UM-CLL and M-CLL subgroups and aggregate data of patient cohorts (12 UM and 11 M) are shown in Figure 2B. The results demonstrate that the proliferation of UM-CLL subgroup is significantly higher than that of M-CLL upon CpG + CD40L stimulation.

The second *in-vitro* proliferation assay utilized the stromal co-culture system described previously [9, 33]. CLL cells were cultured in the presence of stromal NKTert cells and the cell proliferation was assayed after 20-day culture. Figure 2C shows two representative cases from each of the UM-CLL and M-CLL subgroups and aggregate data of patient cohorts (5 UM and 5 M) were shown in Figure 2D. Collectively, these data consistently show that the proliferation in the UM-CLL was higher as compared to the M-CLL subgroup. Taken together, we conclude that UM-CLL cells have higher proliferative capacity than M-CLL.

### UM-CLL is more sensitive to ibrutinib than M-CLL in two *in vitro* proliferation models

**Figure 3:**
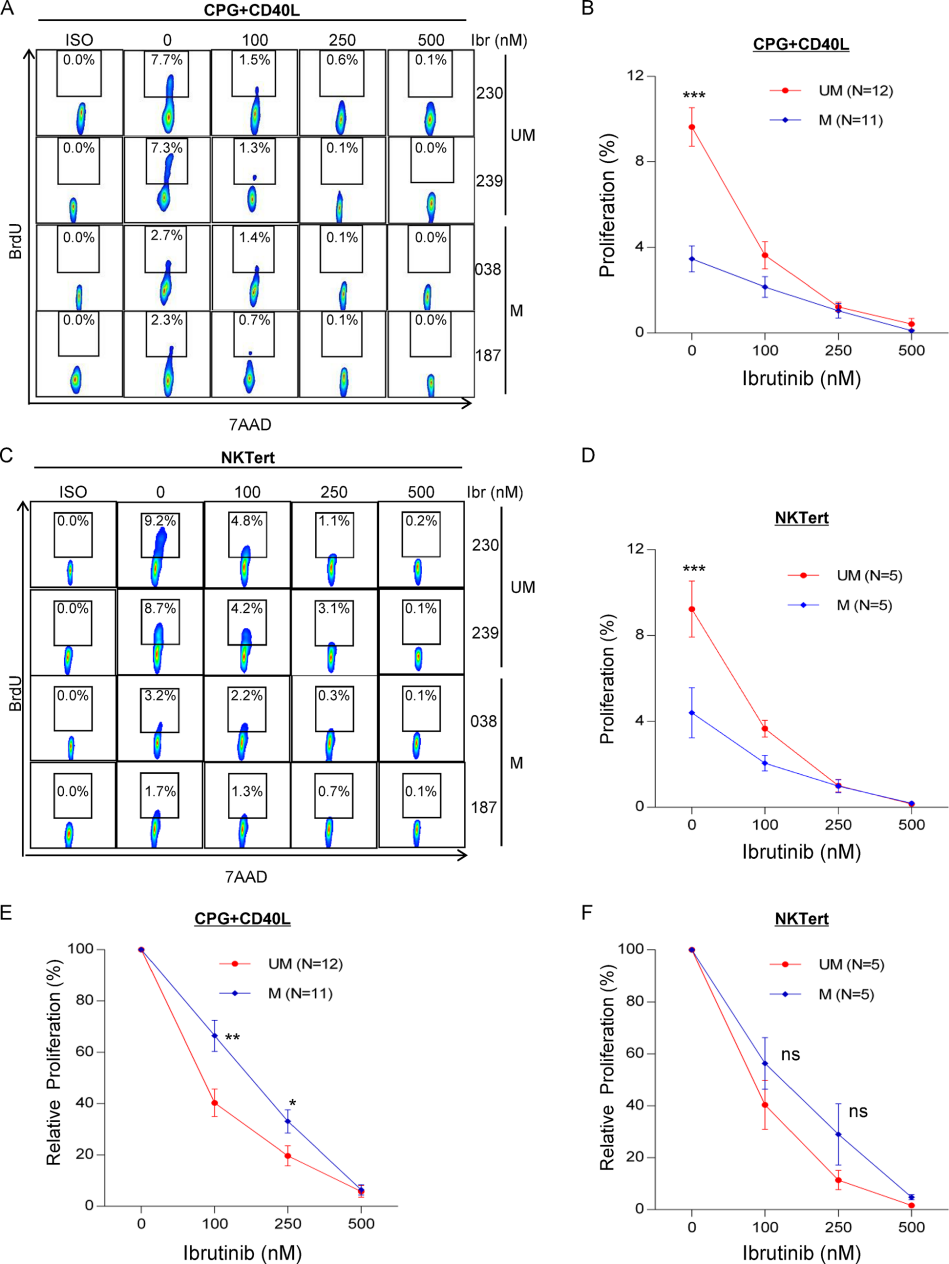
UM-CLL is more sensitive to ibrutinib than M-CLL in both CpG + CD40L and NKTert stromal coculture models (**A, B** and **E**) CpG + CD40L-induced CLL proliferation. BrdU incorporation was measured following 8 days of CpG + CD40L stimulation with or without ibrutinib at indicated concentrations. (**C, D** and **F**) Stroma coculture-induced CLL proliferation. BrdU incorporation was measured following 20 days of ibrutinib treatment at indicated concentrations. (A) Two representative UM-CLL and M-CLL cases are shown. (B) Analysis of the aggregate data of 12 UM-CLL and 11 M-CLL cases. (C) Two representative UM-CLL and M-CLL cases are shown. (D) Analysis of the aggregate data of 5 UM-CLL and 5 M-CLL cases. (E) Relative inhibition of proliferation by ibrutinib under the condition of CpG + CD40L stimulation. Baseline cell proliferation without ibrutinib treatment was normalized to 100%. Analysis was based on raw data presented in Figure 3B. (F) Relative inhibition of proliferation by ibrutinib under the condition of NKTert coculture. Baseline cell proliferation without ibrutinib treatment was normalized to 100%. Analysis was based on raw data presented in Figure 3D. Values in line graphs represent means ± SEM. UM, unmutated CLL. M, mutated CLL. *P < 0.05, **P < 0.01, and ***P < 0.001; ns, not significant.

We have previously shown that BTK inhibition by ibrutinib targets *in vivo* CLL proliferation [9]. We aimed to investigate whether the intrinsic high proliferative capacity makes UM-CLL more vulnerable to ibrutinib treatment. We exposed CpG + CD40L-stimulated CLL cells [34] to various doses of ibrutinb. Figure 3A shows BrdU incorporation in two representative cases from each subgroup and Figure 3B shows data for the cohorts (12 UM and 11 M-CLL). Figure 3B demonstrates a steeper response of UM-CLL to increasing doses of ibrutinib compared with the response of M-CLL. In both subgroups, proliferation was inhibited to the similar low levels at higher concentrations of ibrutinib (≥ 250 nM). These results were validated in the second NKTert stromal coculture model using five UM and five M-CLL (Figure 3C and 3D). In addition, we analyzed these data for relative changes in which the baseline cell proliferation without ibrutinib treatment was normalized to 100%. Figure 3E showed that under the condition of CPG + CD40L stimulation, UM-CLL was significantly more inhibited by 100 and 250 nM of ibrutinib than M-CLL. At a high concentration of 500 nM, cell proliferation was inhibited to a level below 6%, then the differences between UM and M-CLL became insignificant as expected. The same trend was identified with the NKTert model, however, statistical significance was not reached perhaps relating to small number of cases used for this study (Figure 3F). Together, these results confirm our previous observation that BTK inhibition with ibrutinib impairs CLL proliferation [9]. Further, the results suggest that heightened proliferation of UM-CLL makes this group more vulnerable to ibrutinib's action (Also see below).

### The apoptosis effect of ibrutinib does not differ between UM-CLL and M-CLL

**Figure 4:**
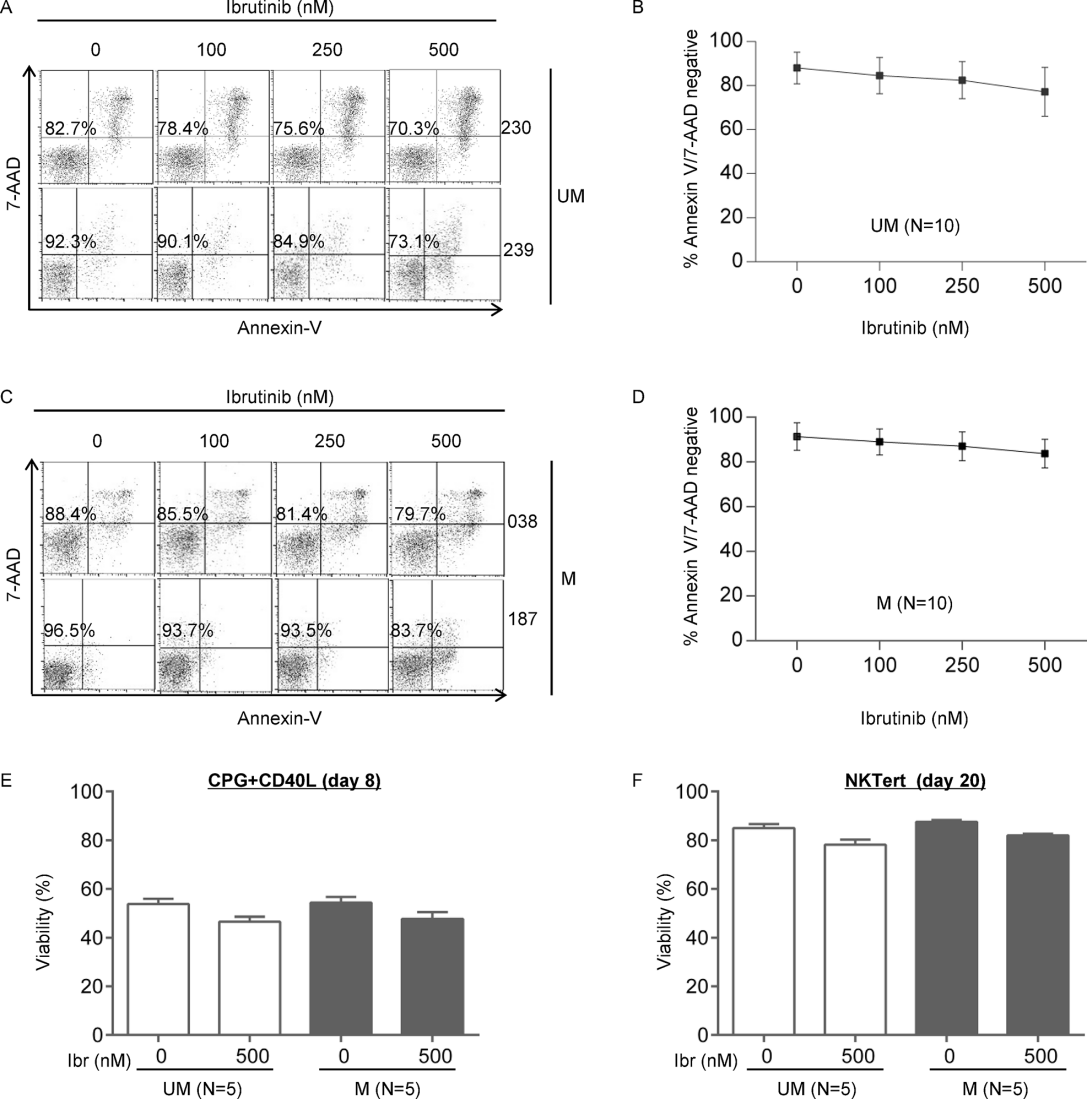
The apoptosis effect of ibrutinib does not differ between UM-CLL and M-CLL Apoptosis of CLL cells was measured using annexin V/7AAD assay following 48 hours of ibrutinib treatment at indicated concentrations. (**A**) Two representative UM-CLL cases are shown. The percentages of viable annexin-V^−^ and 7AAD^−^ cells in the bottom left quadrant were indicated. (**B**) Analysis of the aggregate data of 10 UM-CLL cases. (**C**) Two representative M-CLL cases are shown. The percentages of viable annexin-V^−^ and 7AAD^−^ cells in the bottom left quadrant were indicated. (**D**) Analysis of the aggregate data of 10 M-CLL cases. UM, unmutated CLL. M, mutated CLL. (**E**) Viability of CpG + CD40L stimulated CLL cells at day 8 of culture in the presence or absence of 500 nM of ibrutinib. (**F**) Viability of NKTert-co-cultured CLL cells at day 20 in the presence or absence of 500 nM of ibrutinib.

Several studies suggested that ibrutinib induces apoptosis in CLL, but the effects are minimal to modest [10–12, 37]. We compared apoptotic response of CLL to ibrutinib in UM-CLL versus M-CLL. We measured Annexin V and 7-AAD staining in CLL cells that were treated with increasing doses of ibrutinb. The amount of apoptosis was minimal, less than 10%, both in UM-CLL and M-CLL, even with as high as 500 nM ibrutinib (Figure 4A–4D). The maximal plasma concentration achievable in patients is 408 nM according to a previous pharmacokinetics study [38, 39]. As shown in Figure 4B and 4D, the response curves of UM-CLL versus M-CLL appeared to run in parallel and no discernable differences were observed. The cell viability was also determined in the setting of cell proliferation models with either CpG + CD40L stimulation or NKTert stromal coculture. Again, cell death induced by 500 nM of ibrutinib was minimal suggesting apoptosis induction is not the primary action of ibrutinib (Figure 4E and 4F).

### Heightened proliferation in UM-CLL subgroup is BTK-dependent

**Figure 5:**
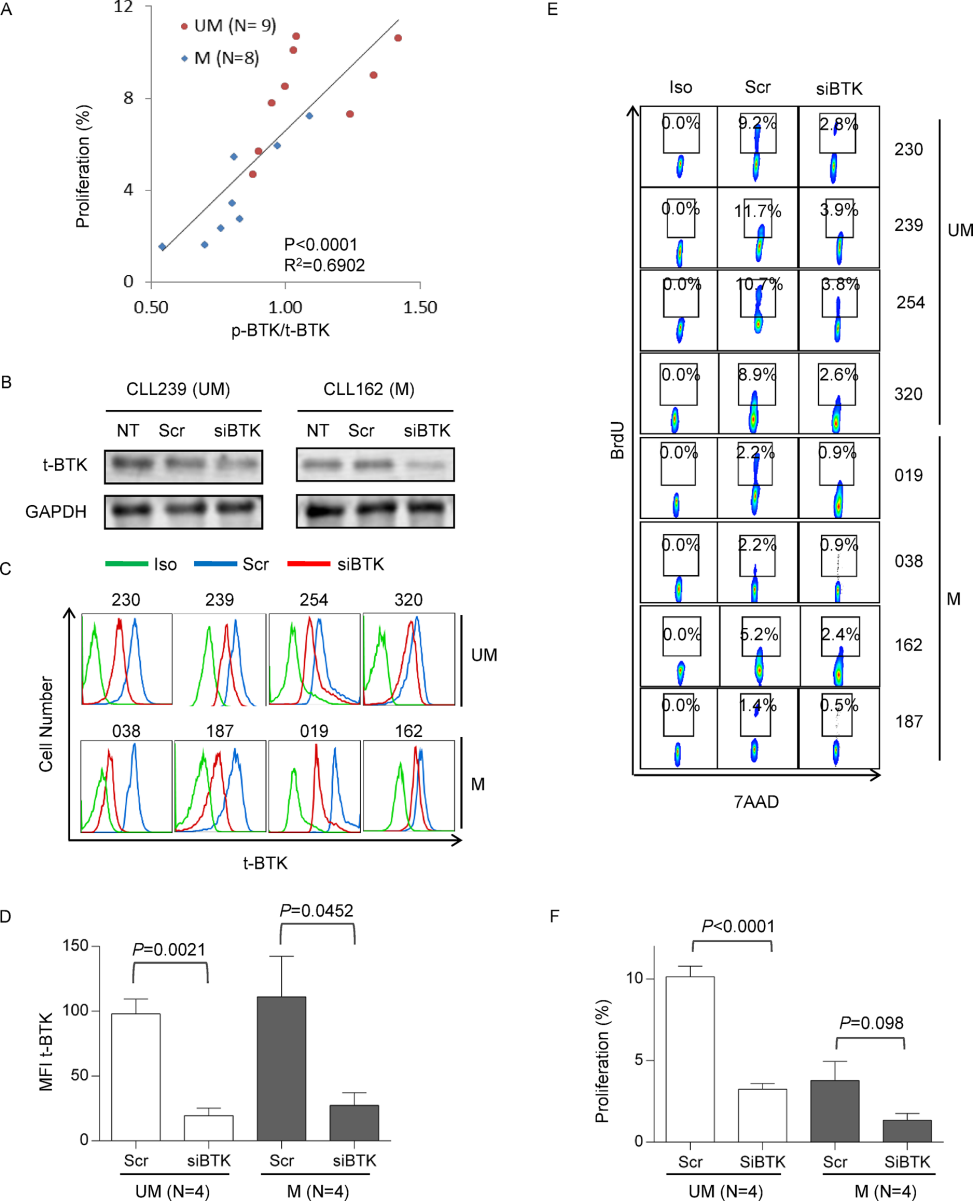
Heightened proliferation in UM-CLL subgroup is BTK-dependent (**A**) Correlation between p-BTK and CLL proliferation. Linear regression of total and statistical analysis was conducted using GraphPad Prism 6. (**B**) Efficiency of BTK knock-down assayed by immunoblotting. CLL cells were transfected with either siBTK or scrambled control siRNAs (*N* = 2). Amounts of BTK were analyzed after 4 days of transfection. (**C**) Efficiency of BTK knock-down assayed by flow cytometry (*N* = 8). Amounts of total BTK were analyzed with anti-BTK antibody after 4 days of transfection. (**D**) Mean fluorescence intensity (MFI) of total BTK were plotted for unmutated (*N* = 4) and mutated CLL patients (*N* = 4). (**E**) Proliferative responses of siBTK- and scrambled RNA-transfected UM- and M-CLL were measured by 7-AAD/BrdU incorporation after 8 days of transfection (*n* = 8). (**F**) Proliferative responses were analyzed with Paired *t* test to compare unmutated and mutated CLL subgroups. Scr, scrambled control siRNAs. siBTK, siRNA against BTK. *P*-values are indicated.

We have demonstrated that UM-CLL, as a group, has higher BTK activity and higher proliferation capacity than M-CLL. We then analyzed whether BTK activity is correlated with CLL proliferation. We plotted ratios of p-BTK/total BTK (Figure 1) against cell proliferation in the absence of ibrutinib treatment (Figure 3A, column 2) for cases that have sufficient number of cells for the conduction of both assays. We found a significant linear correlation between p-BTK levels and proliferation capacity (Figure 5A). Moreover, it becomes apparent that UM-CLL (*n* = 9) has higher p-BTK associated with higher cell proliferation compared to M-CLL (*n* = 8).

To further determine whether CLL cell proliferation depends on BTK, we employed RNA interference technology to knock down BTK. UM-CLL (*n* = 4) and M-CLL (*n* = 4) cells were transfected with either siRNA targeting BTK or scrambled control under the condition of CpG + CD40L stimulation. At day 4 of siRNA transfection, the efficiency of siRNA knock-down was evaluated first with immunoblotting in two cases (Figure 5B) and subsequently with flow cytometry in additional cases (Figure 5C). Significant reduction in total BTK levels was achieved in all samples and to a similar degree in both UM- and M-CLL groups (Figure 5D). Cell proliferation was then determined at day 8 post transfection. Figure 5E shows that BTK knock down by siRNA reduced cell proliferation in all cases. Analysis of UM-CLL and M-CLL showed that the reduction in cell proliferation was much more pronounced in UM-CLL than in M-CLL, due to the higher baseline proliferation in UM-CLL (Figure 5E and 5F). These results are entirely consistent with those obtained with drug inhibition of BTK by ibrutinib (Figure 3). Further, the data indicated that higher BTK activity is one of the major drivers responsible for the higher proliferation in UM-CLL.

### Kinases in the BCR signaling pathways show higher activities and greater ibrutinib responses in UM-CLL compared to M-CLL

**Figure 6:**
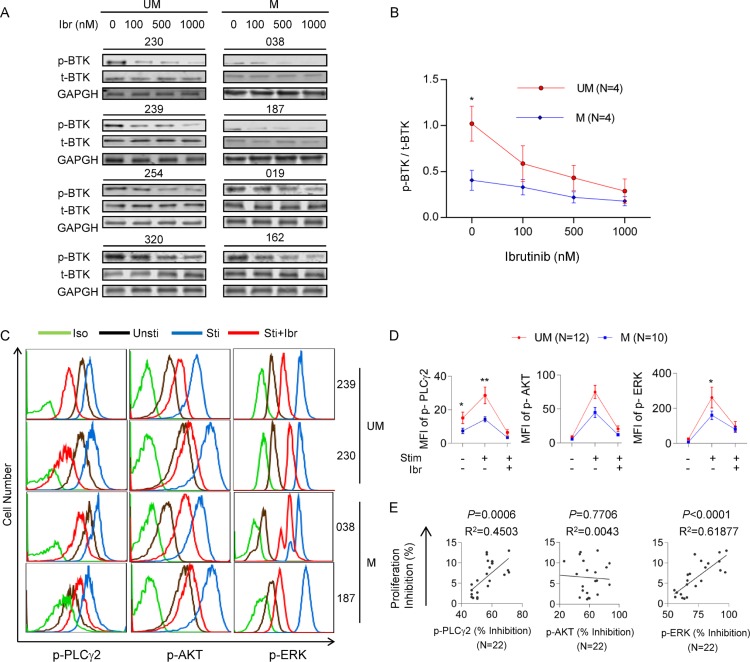
Kinases in the BCR signaling pathways show higher activities and greater ibrutinib responses in UM-CLL compared to M-CLL (**A**) Immunoblotting analysis of p-BTK and t-BTK in CLL cells collected after one hour of ibrutinib treatment at indicated concentrations. GAPDH was used as the loading control. (**B**) Ratios of p-BTK/t-BTK in ibrutinib-treated CLL cells. Band intensities of p-BTK were normalized to the *t*-BTK signals. One-way Anova was used to analyze the dose responses and *t* tests were used to analyze the difference between UM and M-CLL subgroups. Error bars indicate SEM. *P < 0.05. (**C**) Phosphoflow analysis. CLL cells were stimulated with CpG + CD40L for one hour and treated with or without 500 nM of ibrutinib. Simultaneous phospho-flow analyses of p-PLCγ2 (left column), p-AKT (middle column), and p-ERK1/2 (right column) were conducted. Flow analyses of 4 of 22 CLL samples are shown. Iso, isotype control. Unsti, unstimulated cells. Sti, cells stimulated with CpG + CD40L. Sti+Ibr, stimulated cells exposed to 500 nM ibrutinib. (**D**) Mean fluorescent intensity (MFI) of p-PLCγ2, p-AKT, and p-ERK1/2 for each indicated condition were plotted. Twelve unmutated and ten mutated CLL were included in the analyses. (**E**) Percent inhibition of p-PLCγ2, p-AKT and p-ERK1/2 are linearly correlated with the percent inhibition of CLL proliferation. Percent inhibition of protein phosphorylation is defined as [(F_untreated_ − F_treated_)/F_untreated_] × 100%, where F denotes mean fluorescence intensity of 10,000 events. Inhibition of CLL proliferation is defined as the difference between the proliferation rate of untreated cells and the proliferation rate of 500 nM ibrutinib-treated cells (see Figure 3). Data were analyzed using Spearman correlation. *P* values and correlation coefficients are indicated. *P < 0.05, **P < 0.01.

Having established that UM-CLL cells have higher p-BTK levels than M-CLL, we also sought to determine whether higher p-BTK translates into greater sensitivity to ibrutinib inhibition. Four CLL samples from each subgroup were treated with varying doses of ibrutinib under the condition of CpG + CD40L stimulation. As shown in Figure 6A and 6B, compared to M-CLL, the baseline level of p-BTK in UM-CLL is higher and p-BTK decreased more rapidly with increasing concentrations of ibrutinib. Response kinetics of p-BTK to ibrutinib in UM-CLL and M-CLL thus are similar to response kinetics of cell proliferation to ibrutinib (Figure 3), providing another link between BTK activity and cellular proliferation.

To further understand the differential response to ibrutinib between the UM-CLL and M-CLL, we investigated the activities of downstream kinases in the BCR signaling pathways, including PLCγ2, ERK, and AKT. Our group has shown previously that p-PLCγ2, p-ERK and p-AKT were coordinately down-regulated in patients over the course of ibrutinib treatment [9]. We treated UM-CLL (*n* = 12) and M-CLL (*n* = 10) with ibrutinib in the presence or absence of CpG + CD40L stimulation. With phospho-specific flow cytometry, Figure 6C shows four examples in which phosphorylation of PLCγ2, AKT and ERK were up-regulated by the CpG + CD40L stimulation (blue) and down-regulated upon ibrutinib treatment (red). Aggregate data of all samples are shown in Figure 6D. While CpG + CD40L stimulates phosphorylation of PLCγ2, ERK, and AKT in both groups, UM-CLL had a greater response than M-CLL. Further, these elevated kinase activities in UM-CLL subgroup showed a steeper response to ibrutinib inhibition (Figure 6D).

We next studied whether the reduction in kinase phosphorylation correlated with the reduction in cellular proliferation in ibrutinib-treated cells. We analyzed the quantitative relationship between the degree of p-PLCγ2, p-AKT and p-ERK inhibition and percent reduction in cell proliferation as assayed in Figure 3. These analyses revealed a very significant linear correlation of cell proliferation with p-PLCγ2 and with p-ERK, but not with p-AKT, in all 22 CLL cases (Figure 6E). These data suggest that both PLCγ2 and ERK play a key role in mediating the inhibitory effects of ibrutinib on cell proliferation.

## DISCUSSION

The introduction of ibrutinib has significantly improved outcomes for patients with relapsed and refractory CLL, with a majority of patients responding to this well-tolerated oral agent. While mutation status of the IGHV has long been accepted as an important prognostic variable, in the era of ibrutinib and other BCR-targeted therapies, it is unclear whether IGHV will continue to be prognostically relevant. Our investigation sought to compare the differences in molecular/cellular behaviors and pharmacological responses between UM-CLL and M-CLL and explain clinical observations. Our key findings include the following: 1) BTK activity, as reflected by BTK phosphorylation, is significantly higher in UM-CLL than M-CLL; 2) UM-CLL has higher proliferative capacity than M-CLL as demonstrated with two separate *in vitro* models of CLL proliferation; 3) Higher cellular proliferation in UM-CLL made them particularly sensitive to BTK inhibition by ibrutinib; 4) Apoptotic responses to ibrutinib, in contrast, were not different between UM-CLL and M-CLL; 5) Cell proliferation not only correlates with but depends on BTK as demonstrated with BTK depletion using siRNA; 6) Higher BTK activity (p-BTK) in UM-CLL confers greater sensitivity to ibrutinib; Lastly, 7) Higher activities of BCR downstream kinases in UM-CLL render them more sensitive to ibrutinib than M-CLL. These data reinforce each other, and collectively strengthen a linkage between BCR signaling, cell proliferation, and sensitivity to ibrutinib. As opposed to cell proliferation, the results in the current study showed no difference in the rate of apoptosis between UM and M-CLL. These findings extended our recent investigations with both ibrutinib responsive patients and resistant patient showing that ibrutinib acts primarily upon cell proliferation [9, 32–33].

The higher sensitivity of UM-CLL to ibrutinib reported here reflects an inherent greater reliance on BCR signaling for UM-CLL. Using a variety of techniques, several other groups have demonstrated that UM-CLL has higher BCR activity than M-CLL *in vitro* [24–27] that are consistent with our findings showing phosphorylation levels of BTK, PLCγ2, AKT and ERK are uniformly higher in UM-CLL than M-CLL (Figures 5 and 6). Further supporting the differential reliance on BCR signaling are the observations that differential antitumor activity against UM- and M-CLL appears to be commonly shared among several other BCR-directed inhibitors besides ibrutinib. Veldurthy et al. [40] and our group [41] have both reported that dasatinib (primarily targeting LYN) generated a better *in vitro* apoptotic response in UM-CLL than M-CLL. Buchner reported that R408, a SYK inhibitor, also has preferential *in vitro* anti-tumor activity in UM-CLL.[17] In contrast to these studies, only one recent report did not find a correlation between the extent of BCR inhibition and IGVH mutation status in ibrutinib-treated CLL patients.[10] The reason for this discrepant finding is unclear at the present time and warrants further investigation. It remains to be determined whether the BCR-targeted therapies act in clinical trials generating similar or differential responses between the two IGHV subgroups.

Our investigations have several potential limitations. The data were primarily derived from *in vitro* CLL proliferation models. This model was previously validated against an *in vivo* analysis employing samples obtained from ibrutinib-treated patients without *in vitro* culturing [9]. CLL proliferation measured with the model correlates well with patient's clinical sensitivity [9] and clinical resistance to ibrutinib [32, 33]. However, whether the model is valid read-out of CLL proliferation or drug sensitivity in broader settings needs continued investigation. Although data presented here help explain the difference in the overall response rate between UM-CLL and M-CLL, they do not explain why differences between the two groups are lost when partial response with lymphocytosis is added to the response. [20] Based on the previous literature, we suspect that the biological underpinning for peripheral lymphocytosis is more related to the role of BTK in cell adhesion, migration and cytokine signaling [42], and less to its function in BCR signaling as persistent lymphocytes do not show significant up-regulation of BCR-controlled gene expression. [43] Nonetheless, cell adhesion and migration is another important cellular process inhibited by ibrutinib [42, 44, 45]. It cannot not be excluded that the differences between UM and M groups observed here were secondary to their differential expression of CD49d, for example [46].

These investigations have potential clinical implications. With regards to overall response rate to ibrutinib, our mechanistic studies presented here provide molecular insights to explain the differential responses between UM-CLL and M-CLL. In terms of progression-free survival and overall survival, it is questionable whether patients with unmutated IGHV will continue to do worse. The prognostic significance of IGHV mutational status may need to be reevaluated for newer BCR-targeted therapies including, but not limited to SRC, SYK, PI3K and mTOR and other BTK inhibitors.

## MATERIALS AND METHODS

### Patients and healthy donor samples

Peripheral blood samples were collected from 58 CLL patients whose disease diagnosis was based on the clinical and immunophenotypic criteria outlined by IWCLL criteria [47]. Clinical and pathological characteristics of the patients are shown in Supplementary Table 1. These samples were used in various assays described in Figure 1 to Figure 6 based on sample availability. Informed patient consents for the study were obtained according to the Declaration of Helsinki and these studies were approved by the Institutional Review Boards of the University of Chicago and Weill Cornell Medical College. None of the investigated patients had received treatment for at least 3 months prior to sample collection. Progressive and stable disease was defined according to the IWCLL criteria. Among 58 patient samples, 36 were from treatment naïve patients (18 UM-CLL and 18 M-CLL) and these were used to determine the levels of BTK protein expression and phosphorylation in order to avoid any potential impact of prior drug treatment on these measurements (Figure 1).

### Reagents

Ibrutinib was purchased from Selleckchem (Houston, TX, USA), CpG (ODN2006, stimulatory CpG-ODN type B, human specific) was purchased from Invivogen (San Diego, CA, USA), rhIL-4 was ordered from R & D (Minneapolis, MN, USA) and CD40L was obtained from PeproTech (Rock Hill, NJ, USA). Anti-phosphorylated BTK (p-BTK) (Y223) was purchased from Cell Signaling Technology (Danvers, MA, USA); both anti-total BTK antibody and PE-anti-CD38 (clone HIT2) were ordered from BD Biosciences (San Jose, CA, USA) and the GAPDH antibody was procured from Santa Cruz Biotechnology (Santa Cruz, CA, USA); FITC-anti-CD19 (clone HIB19), and PE-anti-CD5 (clone UCHT2) were all purchased from eBioscience (San Diego, CA, USA). Alexa Fluor^®^ 647-anti-phospho-AKT (S473), Alexa Fluor^®^ 488 anti-phospho-p44/42 MAPK (ERK1/2) (T202/Y204) from Cell signaling, or PE-anti-phospho-PLCγ2 (Y759) from BD Bioscience.

### Isolation and *in vitro* culture of CLL cells

CLL cells were purified by using Human B cell Enrichment cocktail Kit from Stemcell Technologies (Vancouver, BC, Canada). The purity of the negatively selected CLL cells exceeded 95% by flow cytometry with CD5+/CD19+ double staining. Isolated CLL cells were then cultured in RPMI-1640 with 15% fetal bovine serum (Gibco, Grand Island, NY, USA), penicillin (100 IU), and streptomycin (100 mg/mL), at a density of 1 × 10^7^ cells/mL in the presence or absence of 2.5 mg/mL CpG plus 500 ng/mL CD40L for various length of time. CLL-NKTert co-culture was conducted as described previously [9, 33].

### Immunoblotting

Whole-cell extracts were prepared by lysing the fresh cells of normal B or CLL cells in RIPA buffer (50 mM Tris–HCl, pH 7.5, 150 mM NaCl, 1% Triton X-100, 0.5% sodium deoxycholate, 0.5% SDS, 2 mM EDTA, 1 × protease inhibitor cocktail II and III and 1 × phosphatase inhibitor). Proteins were separated on 4–12% NuPAGE gel and transferred onto PVDF membranes, incubated with the appropriate primary and secondary antibodies (See Reagents), and subsequently visualized with LI-COR imager (LI-COR biosciences, Lincoln, NE, USA).

### Apoptosis and cell proliferation assays

The cell viability was determined by flow cytometry using the Annexin V/7-AAD Apoptosis Detection Kit I (BD Biosciences) on freshly isolated CLL cells. Bromodeoxyuridine (BrdU) incorporation was analyzed using the FITC BrdU Flow kit (BD Biosciences) following manufacturer's instructions.

### Flow cytometry

Cell staining for FACS analysis was done with optimized amount of fluorochrome-conjugated mAbs as described previously [9, 41, 48]. Briefly, after washing twice with washing buffer (PBS 0.15 M, 0.5% BSA, 0.1% NaN3), 1 × 10^6^ cells were suspended in 100 ml of washing buffer and stained with fluorochrome-conjugated mAbs followed by incubation at 4°C in the dark for 45 minutes. Cells were then washed twice in Perm/Wash buffer before flow cytometric analysis. For intracellular phosphoflow analysis, freshly isolated CLL cells were immediately fixed with 2–4% paraformaldehyde and stored at −80°C. The cryopreserved cells from different CLL samples were simultaneously thawed at room temperature and permeated with 50% methanol on ice for 4 hr following by the staining with specific antibodies. Flow cytometry was conducted using LSR2 flow cytometer (BD Biosciences, San Jose, CA), and the data were analyzed using FlowJo software (Flowjo LLC, Ashland, OR, USA).

### siRNA transfection

siRNA against human BTK was customarily designed and purchased from Thermo Scientific (Waltham, MA, USA). The sense strand sequence of BTK siRNA was: 5′-GUAUGAGUAUGACUUUGAAUU-3′, antisense sequence was: 5′-UUCAAAGUCAUACUCAUAGUU-3′. A non-targeting scrambled siRNA (Scr) was included as the negative control. The siRNA was transfected into CLL cells by using Amaxa cell line Nucleofactor kit V (Lonza, Allendale, NJ, USA). A total of 1 × 10^7^ CLL cells were mixed with 100 μL of Amaxa B-cell nucleofector solution and 2 mg of siRNA. Program X-05 was applied for the electroporation [49].

### Statistical analysis

Student's paired *t*-test or one-way ANOVA was performed to analyze the statistical significance between the groups. Correlations were analyzed using Spearman correlation Method. These statistical analyses were conducted and graphed using Graphpad Prism 6 (GraphPad, La Jolla, CA, USA). *P* values of less than 0.05 were considered significant.

## SUPPLEMENTARY MATERIALS TABLE


