# Supplementary material for: Heightened BTK-dependent cell proliferation in unmutated chronic lymphocytic leukemia confers increased sensitivity to ibrutinib
Source: Oncotarget. 2015 Dec 22;7(4):4598–610. doi: 10.18632/oncotarget.6727 (PMC4826229; doi:10.18632/oncotarget.6727)
Supplement: Supplementary file 1 [file oncotarget-07-4598-s001.pdf]

Heightened BTK-dependent cell proliferation in unmutated chronic lymphocytic leukemia confers increased sensitivity to ibrutinib

Supplementary Materials

Supplementary Table 1: Patients clinical and pathological characteristics

Pt. #	Age	Sex	WBC (× 10^6/mL)	Rai	Therapy	ZAP70	IGHV	CD38	FISH abnormalities*
CLL005	64	M	10	1	NT	Negative	Mut	Negative	del(13q14.3)
CLL008	75	M	10	4	T	Negative	Mut	Negative	del(17p)
CLL011	42	M	23.9	1	T	Positive	Unmut	Positive	Trisomy 12
CLL015	56	F	98.6	1	T	Positive	Unmut	Negative	Trisomy 12
CLL030	60	M	42.5	1	NT	Positive	Unmut	Negative	del(11q22.3), Trisomy 12
CLL033	70	M	11.3	1	NT	Negative	Mut	Negative	del(13q14.3)
CLL037	72	F	84.1	0	NT	Positive	Mut	Negative	del(13q14.3)
CLL038	72	M	58	3	NT	Negative	Mut	Negative	del(13q14.3)
CLL072	79	M	15.8	1	NT	Positive	Mut	Negative	del(13q14.3)
CLL074	41	M	18.2	1	T	Positive	Mut	Negative	del(13q14.3)
CLL082	82	F	22	4	NT	Negative	Mut	Negative	del(11q22.3), del(13q14.3)
CLL085	66	M	11.3	4	T	Positive	Unmut	Negative	del(11q22.3), del(13q14.3), del(17p)
CLL087	79	M	71.5	0	NT	Positive	Mut	Negative	del(17p)
CLL088	87	M	137	4	NT	Positive	Unmut	Negative	del(11q22.3), Trisomy 12
CLL094	62	F	54.8	1	NT	Negative	Mut	Negative	del(13q14.3)
CLL095	80	F	93	1	NT	Positive	Unmut	Negative	del(13q14.3), del(17p)
CLL111	56	F	41.8	1	NT	Positive	Mut	Negative	del(13q14.3)
CLL113	65	F	92	1	T	Positive	Unmut	Negative	normal
CLL116	69	M	85	3	NT	Positive	Unmut	Negative	trisomy 12
CLL117	70	M	62.3	2	T	Positive	Unmut	Negative	del(11q22.3)
CLL124	60	F	29.7	0	NT	Negative	Mut	Negative	del(13q14.3)
CLL131	48	M	19.7	4	T	Negative	Unmut	Positive	del(11q22.3)
CLL143	75	M	89.6	4	NT	Positive	Unmut	Negative	Trisomy 12, del(13q14.3)
CLL153	53	M	23	2	NT	Negative	Mut	Negative	trisomy 12
CLL156	75	F	196	2	NT	Negative	Unmut	Negative	del(13q14.3)
CLL158	54	F	19.4	0	NT	Negative	Unmut	Negative	normal
CLL162	60	M	40	0	NT	Negative	Mut	Negative	del(13q14.3)
CLL179	62	M	28	3	T	Positive	Unmut	Negative	del(11q22.3), del(13q14.3)
CLL187	92	F	48	2	NT	Negative	Mut	Negative	normal
CLL189	60	M	201.3	4	T	Negative	Unmut	Negative	del(11q22.3), del(13q14.3)
CLL195	67	M	299.2	4	T	Positive	Unmut	Negative	del(11q22.3), Trisomy 12, del (13q14.3), del(17p)
CLL208	60	F	103	0	NT	Negative	Mut	Negative	del(13q14.3)
CLL223	62	F	75.6	2	NT	Positive	Mut	Negative	del(13q14.3)

CLL230	66	M	20	4	NT	Positive	Unmut	Positive	del(13q14.3), del(17p)
CLL232	77	M	43.3	1	NT	Positive	Mut	Negative	Trisomy 12
CLL235	66	M	100	4	T	Positive	Unmut	Negative	del(11q22.3), Trisomy 12, del (13q14.3), del(17p)
CLL239	57	M	74.1	4	NT	Positive	Unmut	Negative	del(17p)
CLL248	67	F	65	3	NT	Negative	Mut	Negative	del(13q14.3)
CLL254	55	M	53	1	NT	Negative	Mut	Negative	del(13q14.3)
CLL259	58	M	18	3	NT	Negative	Mut	Negative	del(13q14.3)
CLL262	58	F	95	4	NT	Negative	Unmut	Negative	normal
CLL266	35	M	87.5	4	T	Positive	Unmut	Negative	del(13q14.3)
CLL271	48	F	42	1	NT	Positive	Mut	Negative	del(13q14.3)
CLL275	84	M	88	4	T	Negative	Unmut	Negative	del(11q22.3), Trisomy 12
CLL285	61	M	41	3	NT	Negative	Mut	Negative	normal
CLL282	64	M	55	3	T	Negative	Unmut	Negative	Trisomy 12
CLL288	78	M	67	4	NT	Positive	Unmut	Negative	Trisomy 12
CLL292	77	F	112	4	T	Negative	Unmut	Negative	del(13q14.3), del(17p)
CLL307	62	M	86	3	T	Positive	Unmut	Negative	del(17p)
CLL310	31	F	32.8	2	NT	Negative	Mut	Negative	del(13q14.3)
CLL311	68	M	112	3	T	Negative	Unmut	Negative	Trisomy 12, del(17p)
CLL313	64	M	70	4	T	Negative	Unmut	Negative	del(13q14.3), del(17p)
CLL314	56	M	228	4	T	N/A	Unmut	N/A	N/A
CLL320	65	F	168	4	NT	Positive	Unmut	Negative	del(11q22.3), del(13q14.3)
CLL322	53	F	19	4	T	Positive	Unmut	Negative	trisomy 12
CLL348	55	M	220	4	NT	N/A	Unmut	N/A	N/A
CLL360	47	M	281	2	NT	Negative	Mut	Negative	del(13q14.3)
CLL371	58	M	141	4	T	Negative	Unmut	Negative	Trisomy 12, del(17p)

Abbreviations: CLL, chronic lymphocytic leukemia; F, female; FISH, fluorescence in situ hybridization (only abnormalities detected in ≥ 5% of cells are shown); IGHV, immunoglobulin heavy chain variable region genes; M, male; Mut, mutated; N/A, not available; NT, not treated; T, treated; Unmut, unmutated; WBC, white blood cells count.

*CLL FISH panel: del (11q22.3)(ATM), trisomy 12, del(13q14.3/34), del(17p13.1) (TP53).
